# Editorial: Pediatric Obesity: From the Spectrum of Clinical-Physiology, Social-Psychology, and Translational Research

**DOI:** 10.3389/fped.2021.762189

**Published:** 2021-09-28

**Authors:** Ching-Feng Cheng, Yen-Hsuan Ni

**Affiliations:** ^1^Department of Pediatrics, Taipei Tzu Chi Hospital, Buddhist Tzu Chi Medical Foundation, New Taipei, Taiwan; ^2^Department of Pediatrics, School of Medicine, Tzu Chi University, Hualien, Taiwan; ^3^Department of Pediatrics, National Taiwan University Children's Hospital, Taipei, Taiwan; ^4^College of Medicine, National Taiwan University, Taipei, Taiwan

**Keywords:** pediatrics and adolescents, metabolic syndrome, obesity, school children, NAFLD

Obesity affects nearly one-third of children in the United States with nearly 6% of adolescents in severely obese condition and is currently a growing global epidemic that requires attention. Increasing expenses in the pediatric health care system were spent in obese children vs. children with a normal body mass index (BMI). Pediatric obesity is also noted to connect with an increased risk of many diseases such as diabetes, cardiovascular diseases, metabolic syndrome, non-alcoholic fatty liver disease (NAFLD), social and psychological problems in adolescents and young adults, and even certain types of cancer later in life. A good outline for childhood and adolescent obesity was presented by Kansra et al., spointing out that the most common cause of obesity throughout childhood and adolescence is an inequity in energy balance; that is, excess caloric intake without appropriate caloric expenditure. They give an overview from the spectrum of clinical-physiology, social-psychology, pathophysiology, and treatments options for obese pediatric and adolescent patients.

In adolescent obesity and health medicine, retrospective cohort studies in school children and anthropometric measurements affecting BMI and obesity were reported. Firstly, Hsiao et al. from Taiwan had compared the growth velocity with BMI. They found that obese and overweight girls from 9 to 13-year-old had less linear growth whereas puberty may dominate over BMI as the main contributor to high growth velocity in pre-puberty girls with underweight BMI. Secondly, dos Santos et al. from Portugal has established predictors of BMI trajectories using anthropometric measurements. They found that weight trajectories were mainly settled by early adolescence. Lack of sleep and eating routines, low emotional self-regulation, child-parent conflict, and low child-parent closeness in early childhood were significantly associated with unhealthy weight trajectories in adolescent and even adult life. Children and adolescents with obesity may also show increased lower urinary tract symptoms, such as urgency and enuresis (Wang et al.), and increased prevalence of hematuria/ proteinuria in school children (Chen et al.), although the etiology is currently unclear and may associate with sex, age, and even urbanization.

The prevalence of metabolic syndrome in children and adolescents is increasing, in parallel with the increasing trends in obesity rates. The cardinal features in pediatric metabolic syndrome included overweight and obesity, abnormal glucose metabolism, dyslipidemia, and hypertension. Other disorders associated with metabolic syndrome include fatty liver, NAFLD, and pro-inflammatory states. A cross-sectional study conducted by Valle-Martos et al. showed that 6–9 years-old school children with obesity, compared to children with normal weight, had higher values for liver enzymes, leptin, markers of insulin resistance, and inflammation, and endothelial dysfunction, and variables associated with metabolic syndrome.

NAFLD has become a public health issue in obese children and adolescents and the clinical course can be progressive and become chronic if not detected at an early stage. There are three reports in our current Topic covering the genetic, histology, and potential treatment in pediatric NAFLD, respectively. Recently, NAFLD was considered to be the hepatic component of metabolic syndrome, and therefore redefined as metabolic associated fatty liver disease (MAFLD). Lin et al. described the new perspectives on genetic prediction. Genetic variants including PNPLA3, TM6SF2, GCKR, MBOAT7, and HSD17B13 have been shown to confer susceptibility to MAFLD in children. Lee et al. elaborated on the relationship between histological features of NAFLD and ectopic fat on MRI in children and adolescents. The third article from Al-Baiaty et al. demonstrated possible hepatoprotective effects in using vitamin E with a high content of tocotrienol as a therapeutic alternative in treating NAFLD in obese children and adolescents.

Recent research findings has significantly increased our understanding of the mechanisms involved in white adipose tissue (WAT) browning and brown adipose tissue (BAT) activation. The discovery of metabolically active BAT in human adults, especially in lean people after cold exposure, has provoked the “thermogenic anti-obesity” idea to battle weight gain. Lu et al. clearly elaborated on the molecular mechanisms regulating UCP1 expression and discussed the potential and caution in targeting UCP1 for enhancing thermogenesis as a strategy to combat obesity.

In summary, this Research Topic has comprehensively covered important aspects of pediatric obesity research from the spectrum of clinical-physiology, social-psychology, and translational research.

## Author Contributions

All authors listed have made a substantial, direct and intellectual contribution to the work, and approved it for publication.

## Funding

This work was support by Buddhist Tzu Chi Medical Foundation (TCMMP108-04-02 and TCAS-108-01) to C-FC.

## Conflict of Interest

The authors declare that the research was conducted in the absence of any commercial or financial relationships that could be construed as a potential conflict of interest.

## Publisher's Note

All claims expressed in this article are solely those of the authors and do not necessarily represent those of their affiliated organizations, or those of the publisher, the editors and the reviewers. Any product that may be evaluated in this article, or claim that may be made by its manufacturer, is not guaranteed or endorsed by the publisher.

